# The complete chloroplast genome of *Melastoma dendrisetosum* (melastomataceae), a shrub endemic to Hainan island, China

**DOI:** 10.1080/23802359.2026.2746834

**Published:** 2026-10-10

**Authors:** Hongxin Wang, Yongxing He, Langxing Yuan, Yiran Liu

**Affiliations:** a Zhai Mingguo Academician Work Station, University of Sanya, Sanya, Hainan, China; b Sanya Nanfan Research Institute, Hainan University, Haikou, Hainan, China; c Tropical Crops Genetic Resources Institute, Chinese Academy of Tropical Agricultural Sciences, Haikou, Hainan, China

**Keywords:** *Melastoma dendrisetosum*, genome characterization, phylogenetic analysis

## Abstract

*Melastoma dendrisetosum* is a narrowly distributed shrub endemic to Hainan, China, and lacks published genomic data. We sequenced, assembled, and annotated its complete chloroplast genome. The plastome is 156,649 bp in length, exhibits a typical quadripartite structure, and contains 131 annotated gene copies (87 protein-coding, eight rRNA and 36 tRNA genes) representing 112 non-redundant genes. Maximum-likelihood and Bayesian analyses of 20 complete chloroplast genomes consistently resolved *M. dendrisetosum* as sister to *M. candidum* with strong support. This study provides valuable genomic resources for future phylogenetic, taxonomic, and conservation studies of the genus *Melastoma*.

## Introduction

The genus *Melastoma* L. 1753 (Melastomataceae) comprises approximately 100 species distributed widely in tropical and subtropical regions across Asia and Oceania (Meyer 2001). Many taxa within this genus possess ethnomedicinal value and are widely used by local communities, predominantly in Southeast Asia, to treat bleeding, diarrhea, diabetes, and gynecological tumors (Zheng et al. 2021). Frequent interspecific hybridization and pervasive gene introgression have blurred species boundaries within the genus, and this taxonomic confusion cannot be resolved by morphology or short nuclear gene fragments alone (Liu et al. 2014).

*Melastoma dendrisetosum* C. Chen 1983 is an endemic shrub narrowly distributed in eastern Hainan Province, China (Figure 1). It was first described from Hainan material but was later reduced to synonymy under *Melastoma sanguineum*; morphological, micromorphological and multi-locus nuclear evidence supports its recognition as a distinct species, and its narrow range and low genetic diversity pose severe conservation challenges (Liu et al. 2014; Huang et al. 2018; Wen et al. 2020).

**Figure 1.**
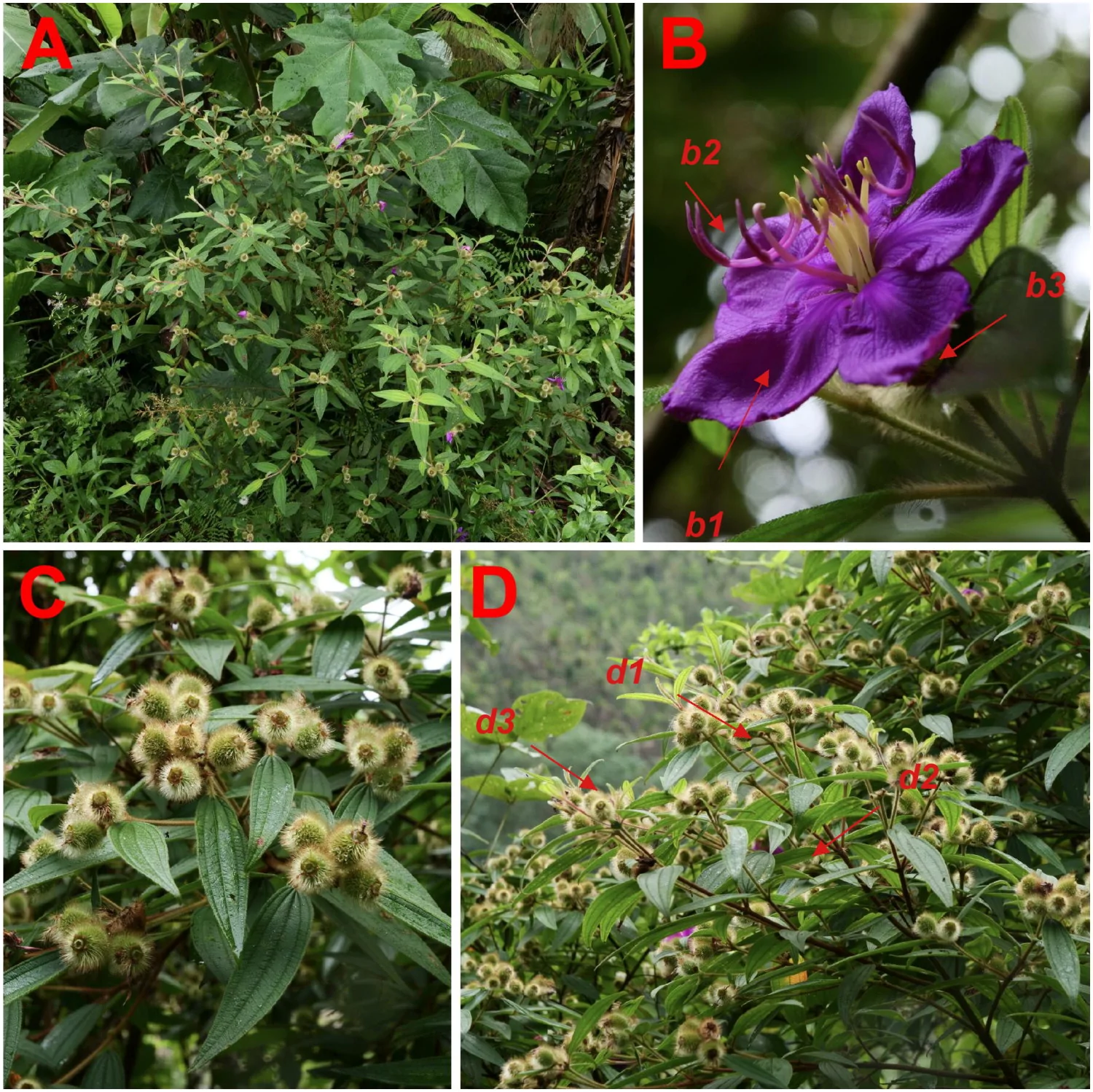
*Melastoma dendrisetosum* C. Chen. (A) Habit of a fruiting individual. (B) Flower, showing the five rhombic-obovate petals with an apical tuft of bristles and shortly ciliate margins (***b1***), the long stamens with an elongated, curved connective base bearing two small tubercles at the apex (***b2***), and the hypanthium densely covered with white, basally branching bristles (***b3***). (C) Close-up of young fruits and persistent calyces densely covered with branched bristles. (D) Fruiting branches and leaves, showing the urceolate, truncate young fruits densely covered with white, basally branching bristles (***d1***), the lanceolate leaves with five basal veins and bristly-ciliate margins (***d2***), and the young branches densely covered with white silky hairs (***d3***). Photographs supplied by the authors; locality: Huishan town, qionghai city, Hainan province, China.

Maternally inherited plastid sequences are less affected by hybridization and introgression and thus provide reliable markers for species discrimination and plastid phylogenetics (Daniell et al. 2016). To date, complete chloroplast (cp) genomes have been published for several congeneric species of *Melastoma* (Ng et al. 2017; Zheng et al. 2019), but none for the critically endangered *M. dendrisetosum*. This study aimed to characterize its plastome and to clarify its phylogenetic position among all publicly available *Melastoma* plastomes.

## Materials and methods

### Plant material

Fresh leaves were collected from a single individual in Huishan Town, Qionghai City, Hainan Province, China (19°05′35.2ʺN, 110°15′47.7ʺE). The specimen was identified as *Melastoma dendrisetosum* by Langxing Yuan, and the leaves were dried in silica gel for DNA extraction. A voucher specimen (YMD20241005) was deposited at the Herbarium of the Institute of Tropical Crop Germplasm Resources, Chinese Academy of Tropical Agricultural Sciences (https://www.ctcgris.cn/en; contact: Langxing Yuan, orange269@163.com).

### DNA extraction and sequencing

Total genomic DNA was extracted from silica-dried leaves using a modified CTAB method (Doyle and Doyle 1987). Prior to library construction, the A260/A280 ratio (approximately 1.7) and the DNA concentration (approximately 20 ng/µL; Qubit 3.0 fluorometer, Thermo Fisher Scientific, Waltham, MA, USA) were assessed, and integrity was checked by 1% agarose gel electrophoresis. A paired-end library (approximately 300-bp insert) was sequenced as PE150 reads on a DNBSEQ-T7 instrument (MGI Tech Co., Ltd., Shenzhen, China). Fastp v0.24.0 removed adapters and low-quality data and retained reads at least 140 bp long; other settings were default (Chen et al. 2018).

### Assembly and annotation of the chloroplast genome

The complete cp genome was de novo assembled using GetOrganelle v1.7.7.1 with parameters -F embplant_pt -t 10 (Jin et al. 2020). This pipeline calls SPAdes v4.2.0 with k-mer values of 21, 55, 85 and 115 to perform de novo assembly (Bankevich et al. 2012), and the complete circular plastome graph was visually verified in Bandage v0.8.1 (Wick et al. 2015). Annotation was performed using Geseq (Tillich et al. 2017), referencing *Melastoma candidum* (KY745894), *Melastoma penicillatum* (NC_077578), and *M. sanguineum* (NC_077579). The circular plastome map was generated in OGDRAW v1.3.1, and cis- and trans-spliced gene structures were displayed with CPGView (Greiner et al. 2019; Liu et al. 2023).

### IR boundary analysis

Comparisons between the boundaries of three regions (large single-copy (LSC), small single-copy (SSC), and inverted repeats (IRs)) were generated using IRscope v0.1 (Amiryousefi et al. 2018).

### Phylogenetic analysis

To determine the phylogenetic placement of *M. dendrisetosum*, we integrated its cp genome with 19 previously released cp genomes of congeneric taxa for comparative analyses.The 20 cp genomes were aligned using MAFFT v7.525 (Katoh and Standley 2013). Maximum-likelihood (ML)analysis was performed in IQ-TREE v3.0.1 under the K3Pu + F + I model selected by ModelFinder, with 1,000 SH-aLRT and 1,000 ultrafast-bootstrap replicates (Anisimova et al. 2011; Kalyaanamoorthy et al. 2017; Hoang et al. 2018; Wong et al. 2026). Bayesian inference (BI) was conducted in MrBayes v3.2.7 under GTR+F + I + G4 using two runs of four chains for 2,000,000 generations, with 25% burn-in (Ronquist et al. 2012). Convergence was satisfactory (ASDSF = 0.001542; PSRF = 1.000–1.002; ESS > 443). The outgroups *Pternandra korthalsiana* and *Pternandra tessellata* were designated to root the phylogenetic trees. All resulting topologies were graphically edited and visualized using FigTree v1.4.4 (Rambaut 2018).

## Results

### Plastome features

The complete cp genome of *M. dendrisetosum* is 156,649 bp, comprising an LSC region of 86,059 bp, an SSC region of 17,086 bp and a pair of IR regions of 26,752 bp, with a total GC content of 37.2% (Figure 2). The average sequencing depth of the assembly was 803× (Figures S1 and S2). After excluding the two outgroups, the 18 ingroup cp genomes ranged from 155,103 bp (MK994885) to 156,695 bp (NC_077578) (Table S1).

**Figure 2.**
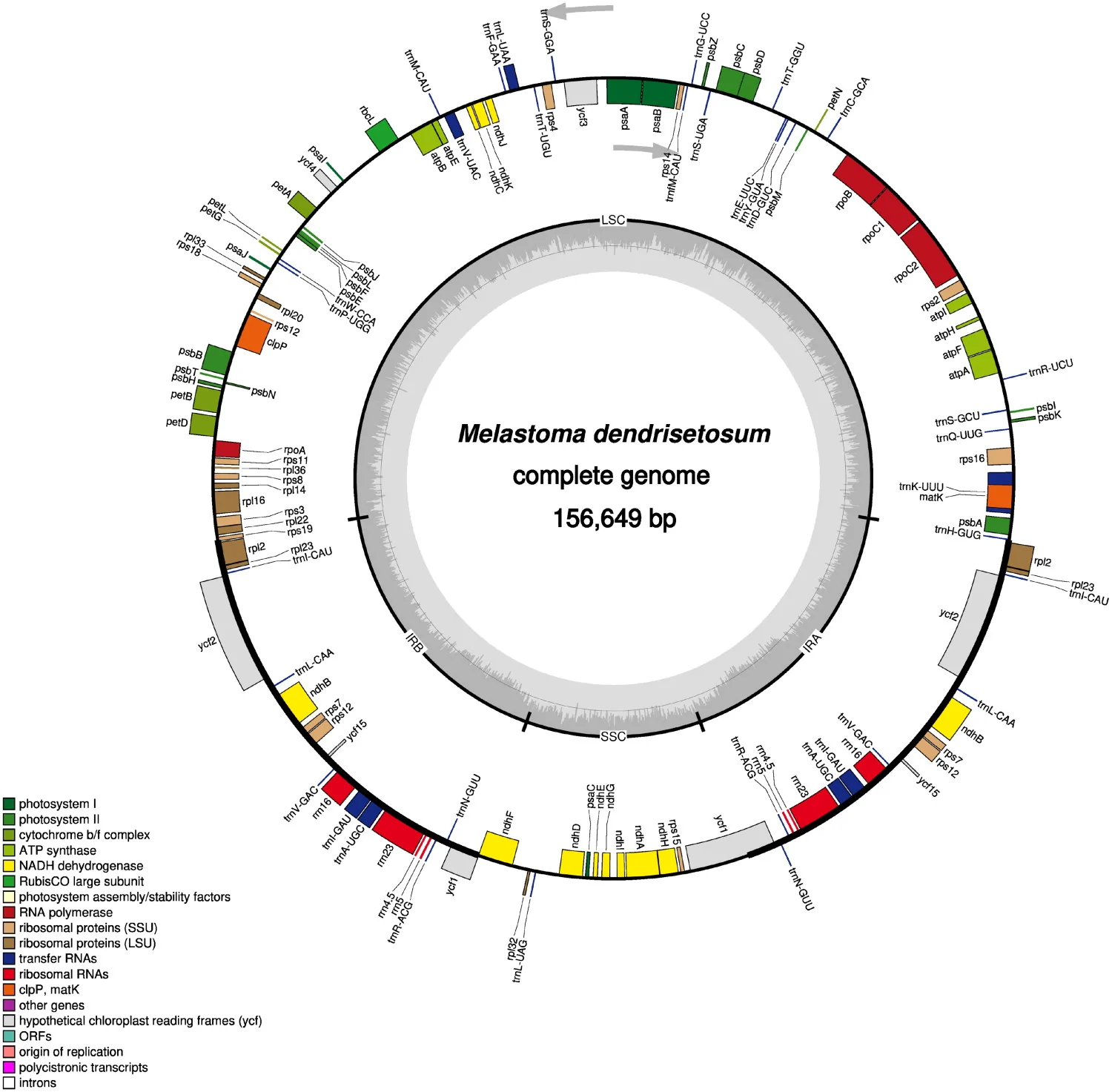
Circular map of the complete chloroplast genome of *melastoma dendrisetosum*. Genes drawn outside and inside the circle are transcribed counterclockwise and clockwise, respectively. Colors denote functional categories. The inner gray plot represents GC content, and the boundaries of the large single-copy (LSC), small single-copy (SSC) and inverted-repeat regions (IRa and IRb) are indicated.

### Gene content

The complete cp genome contained 131 annotated gene copies, including 87 protein-coding, 36 tRNA and eight rRNA genes, representing 112 non-redundant genes, of which 18 were duplicated in the IR regions and 17 contained introns, plus one truncated *ycf1* pseudogene copy (ψ*ycf1*) spanning the IRb/SSC junction (Table S2). Two genes (*clpP* and *ycf3*) contained two introns each (Figure S3), and *rps12* was trans-spliced (Figure S4).

### IR boundary analysis

The cp genome structures of the six *Melastoma* species were highly similar (Figure S5). The *ndhF* gene extended 40 bp into the IRb and the 5′ portion of *ycf1* in the SSC was 3,724 bp in all six species, whereas the main variation occurred at the LSC/IRb boundary, where *rps19* extended 79 bp into the IRb in *M. dendrisetosum*, *M. malabathricum* and *M. candidum*, but only 7 bp in the other three species. No obvious expansion or contraction of the IR regions was detected among the six plastomes.

### Phylogenetic position

ML and BI analyses produced identical topologies (Figure 3 and Figure S6). Three major, well-supported clades were recovered: Melastomateae (Renner 1993), Dissochaeteae (Clausing and Renner 2001; Kartonegoro et al. 2018), and the outgroup. The tribe Melastomateae formed a strongly supported monophyletic clade. Within this clade, *M. dendrisetosum* was strongly supported as sister to *M. candidum*; this pair was successively sister to *M. penicillatum, M. sanguineum*, and the clade comprising *Melastoma malabathricum* and *Melastoma dodecandrum,* all with robust support. The remaining Melastomateae genera (*Heterotis*, *Osbeckia* and *Heterocentron*) formed a well-supported subclade, and Dissochaeteae was robustly monophyletic, with *Medinilla* and *Heteroblemma* as distinct subclades.

**Figure 3.**
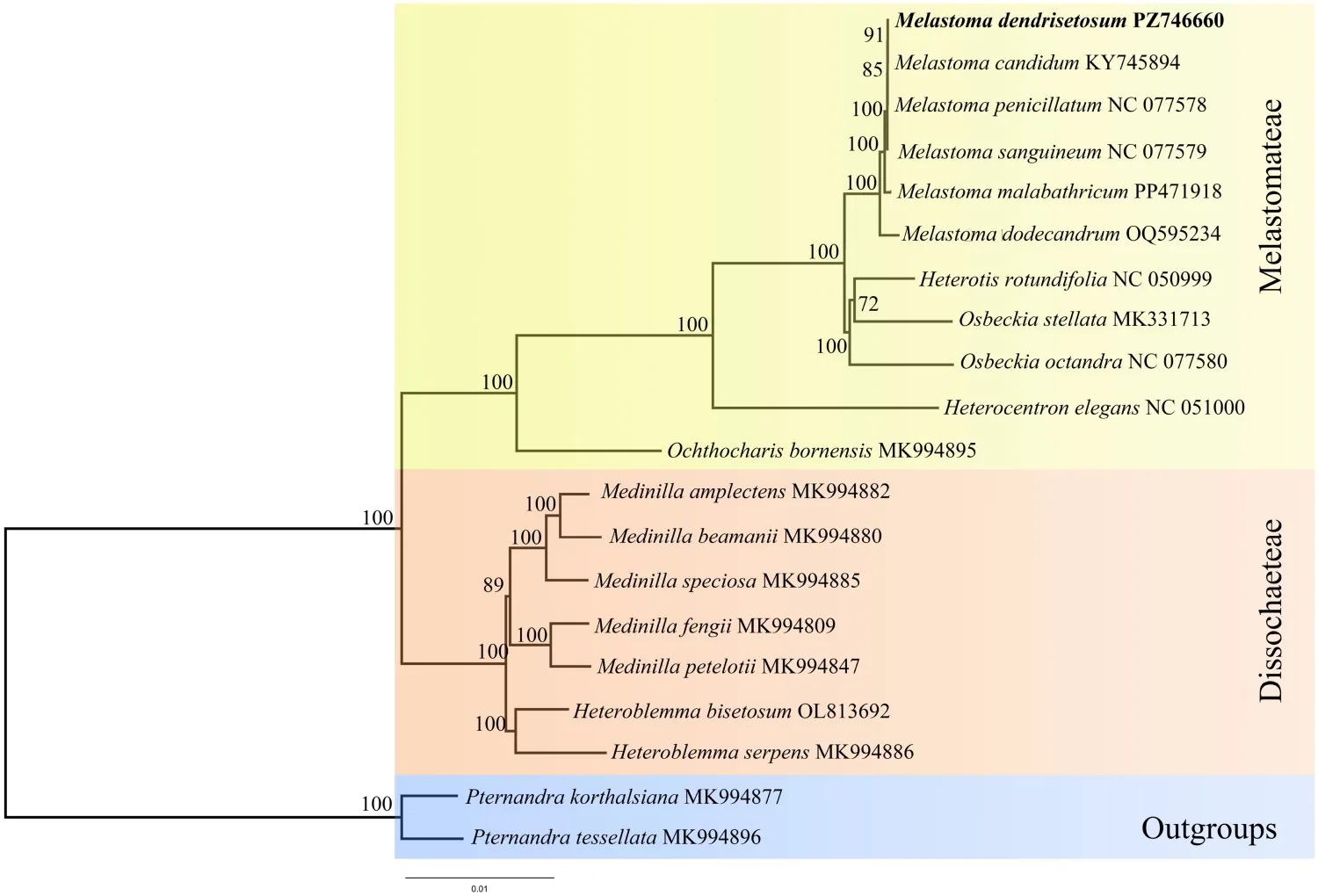
Maximum likelihood phylogenetic tree inferred from 20 chloroplast genomes using the whole length, *pternandra korthalsiana* and *pternandra tessellata* were used as the outgroups. The sample with black font was the chloroplast genome of *melastoma dendrisetosum*. The following sequences were used: *Melastoma dendrisetosum* PZ746660 (this study), *M. candidum* KY745894 (Ng et al. 2017), *M. penicillatum* NC_077578 (Huang et al. 2023), *M. sanguineum* NC_077579 (Huang et al. 2023), *M. malabathricum* PP471918(Huang et al. 2023), *M. dodecandrum* OQ595234 (Huang et al. 2023), *Heterotis rotundifolia* (Zhang et al. 2020)*, Osbeckia stellata* MK331713 (Liang et al. 2019), *Osbeckia octandra* NC_077580 (Huang et al. 2023), *Heterocentron elegans* NC_051000 (Zhang et al. 2020), *Ochthocharis bornensis* MK994895 (Zhou et al. 2019), *Medinilla amplectens* MK994882 (Priyadi et al. 2024), *Medinilla beamanii* MK994880(Priyadi et al. 2024), *Medinilla speciosa* MK994885 (Zhou et al. 2022), *Medinilla fengii* MK994809 (Zhou et al. 2022), *Medinilla* petelotii MK994847 (Zhou et al. 2022), *Heteroblemma bisetosum* OL813692 (Zhou et al. 2022), *Heteroblemma serpens* MK994886 (Zhou et al. 2022), *Pternandra korthalsiana* MK994877 (Zhou et al. 2022), *Pternandra tessellata* MK994896 (Zhou et al. 2022).

## Discussion and conclusion

Among the representative *Melastoma* cp genomes listed in Table S1, M. *dendrisetosum* shares nearly identical genome size, LSC/SSC/IR dimensions, quadripartite architecture and gene inventory with previously published Melastomataceae plastomes, reflecting the universal structural conservation of this family (Reginato et al. 2016; Ng et al. 2017; Zheng et al. 2019).

The plastid tree clearly separated the two Hainan endemic taxa from other congeneric lineages, with *M. dendrisetosum* sister to *M. candidum*, while *M. penicillatum* occupied an independent branch within the core *Melastoma* group. Because widespread hybridization and plastid capture occur across Melastoma and readily cause cytonuclear discordance (Huang et al. 2018), cytoplasmic signals must be interpreted cautiously. Nuclear multi-gene evidence nevertheless validates M. dendrisetosum as an evolutionarily independent species, with no shared nuclear haplotypes with sympatric congeners such as *M. sanguineum* and *M. penicillatum*, and its hypanthial indumentum consistently distinguishes it from *M. penicillatum* (Huang et al. 2018). Thus, although the maternally inherited plastome records only the maternal history, our tree, congruent with diagnostic morphology and nuclear data, clearly identifies the closest plastid relatives of the two Hainan endemic shrubs. Given the rapid recent radiation and blurred species boundaries in *Melastoma*, complete cp genomes markedly improve phylogenetic resolution and species delimitation.

In conclusion, we report the complete cp genome of *M. dendrisetosum* and clarify its phylogenetic placement within *Melastoma*, providing genomic resources for comparative plastomics, barcode screening, population genomics and conservation of this endangered Hainan endemic shrub.

## Supplementary Material

Supplemental Material

Supplemental Material

## Data Availability

The genome sequence data supporting this study are openly available in GenBank of NCBI at https://www.ncbi.nlm.nih.gov under the accession number PZ746660. The associated BioProject, SRA, and BioSample numbers are the following: BioProject (PRJNA1501936), SRA (SRR39834775), and BioSample (SAMN61961214), respectively.
