# Supplementary material for: The complete chloroplast genome of Melastoma dendrisetosum (melastomataceae), a shrub endemic to Hainan island, China
Source: Mitochondrial DNA B Resour. 2026 Oct 10;11(11):1306–11. doi: 10.1080/23802359.2026.2746834 (PMC13656650; doi:10.1080/23802359.2026.2746834)
Supplement: Supplementary file 1 [file TMDN_A_2746834_SM3427.docx]

Table S1. The total, LSC, SSC, and IR region length of chloroplast genomes used in this study.

| **Species** | **Accession number** | **Total length (bp)** | **LSC (bp)** | **SSC (bp)** | **IR (bp)** |
| --- | --- | --- | --- | --- | --- |
| *Melastoma dendrisetosum* | PZ746660 | 156,649 | 86,059 | 17,086 | 26,752 |
| *Melastoma candidum* | KY745894 | 156,682 | 86,084 | 17,094 | 26,752 |
| *Melastoma penicillatum* | NC_077578 | 156,695 | 86,078 | 17,093 | 26,762 |
| *Melastoma sanguineum* | NC_077579 | 156,671 | 86,082 | 17,099 | 26,745 |
| *Melastoma malabathricum* | PP471918 | 156,606 | 86,008 | 17,082 | 26,758 |
| *Melastoma dodecandrum* | OQ595234 | 156,610 | 86,015 | 17,095 | 26,750 |
| *Heterotis rotundifolia* | NC_050999 | 156,336 | 85,737 | 17,121 | 26,739 |
| *Osbeckia stellata* | MK331713 | 156,372 | 85,741 | 17,115 | 26,758 |
| *Osbeckia octandra* | NC_077580 | 156,084 | 85,990 | 17,182 | 26,756 |
| *Heterocentron elegans* | NC_051000 | 156,514 | 85,944 | 17,162 | 26,704 |
| *Ochthocharis bornensis* | MK994895 | 156,672 | 86,033 | 17,101 | 26,769 |
| *Medinilla amplectens* | MK994882 | 155,529 | 85,372 | 16,524 | 26,813 |
| *Medinilla beamanii* | MK994880 | 155,271 | 85,025 | 16,633 | 26,803 |
| *Medinilla speciosa* | MK994885 | 155,103 | 84,787 | 16,752 | 26,782 |
| *Medinilla fengii* | MK994809 | 155,841 | 85,629 | 16,632 | 26,790 |
| *Medinilla petelotii* | MK994847 | 155,673 | 85,355 | 16,712 | 26,803 |
| *Heteroblemma bisetosum* | OL813692 | 156,124 | 85,703 | 16,853 | 26,784 |
| *Heteroblemma serpens* | MK994886 | 156,047 | 85,661 | 16,828 | 26,779 |
| *Pternandra korthalsiana* | MK994877 | 157,496 | 86,730 | 17,358 | 26,661 |
| *Pternandra tessellata* | MK994896 | 156,994 | 86,249 | 17,333 | 26,749 |
